# Account of Diseases in an Eastern District of London, from the 20th of October to the 20th of November

**Published:** 1799-12

**Authors:** 


					Shite of Difiafes in London. 4.13
Account of Dlfeafes in an Eajiern D if rid of London, frcm the 20th of
Oiiober to the zotb cf 2Jo-vember.
No. of Cafes.
ACUTE DISEASES.
Typhus Gravior - - 3
Typhus Mitior - 6
Scarlatina 2
Scarlatina Anginofa - 3
Peripneumonia 3
Acute Rheumatifm - -2
.CHRONIC DISEASES.
Peripneumonia notha - 8
Cough - - 12
Dyfpncea 9
Cough and Dyfpncea - 14
Phthifis Pulmonalis - 5
Hajmoptoe - - - ?
Hydrothorax - - 2
Palpitatio 2
Apoplexia - - -2
No. of Cafes.
Paralyfis - - I
Epilepfia -- I
Amentia I
Dyfpepfia - 8
Vomitus - - - 3
Diarrhoea - - 16
Dyfenteria - - ? - 4
Colica 3
Coljca Pittonum - z
Jnteftinal Hainiorhagy - 1
Hepatalgia - - - I
Nephralgia 1
Amenorrhea - - 6
Chlorofis - ~ 9
Hyfteria 4
Chronic Rheumatifm - 12
FUER-
* Dr. Cullaclcn.
4T4 State of D ifeafes in London.
PUERPERAL DISEASES.
Ephemera - - 6
Menorrhagia Iochialis - 3
Dolor poll partum - 2
Rhagas papilla; - ,2
INFANTILE DISEASES.
Meafles - ' - 6
Hooping Cough - - 7
Tabes Mefenterica - 2
Scrophula 2
Since the laft report, there has been a train of difeafes fimilar to thofe
which were then taken notice of. Inteftinal complaints continue to
form a principal fhare of the lift. The greateft number of thefe have
proved rather tedious and troublefome than violent and alarming. Th
mealies, which have, for fome time, prevailed amongft children, occur
lefs frequently. This difeafe is likely to be fucceeded by fcarlatina, of
which there are at prefent feveral inftances.
It has hitherto appeared in a mild form. In fome cafes, the fcarlct
eruption has been attended with very flight affections of the throat, and
the difeafe has very much refembled that which was defcribed by Sy-
denham, and which, he obferves, generally makes its appearance at the
clofe of fummer. The exiltence of the diieafe in this mild form, as
noticed by Sydenham, has been queftioned by fome who have been
always accufcomed, to confider the affedlions of the throat as a ne-
ceflary charafteriftic of the difeafe. Others have fpoken as confi-
dently of the exiftence of it, as defcribed by him, where the angi-
nofe fymptoms, if they exiiled, were fo flight as not to form a pro-
minent fymptom.
That this fymptom did not form a part of the difeafe to which he
refers may be taken for granted, when we recollett how acute his obfer-
vation, and how accurate his defcription, of difeafe ; but it is equally
certain that, fince his time, this fymptom, in a more or lefs evident de-
gree, has generally accompanied the complaint.
'When children have been the fubje&s of this difeafe, it has more fre-
quently appeared in its fimple form, than when adults have been the
fubjefts of it; and this circumftance ferves to reconcile the observation
of Sydenham with v/hat takes place at prefent,?that, though it feizes
whole families, children are more particularly liable to it.
This difeafe, as was before remarked, appeared in a mild form, in
mpft of the inftances referred to in the lift. In one patient, however, a
child of four years of age, the fymptoms were more aggravated; the
tonfils were coniiderably enlarged and inflamed; deglutition was diffi-
cult; a large fscretion of tough mucus throughout the fauces, occafioned
a dif-
a difficulty of breathing, and a material change in the voice. All thefe
fymptoms were relieved by external fuppuration taking place, and the
patient Toon recovered.

				

